# Blast-Induced Mild Traumatic Brain Injury Alterations of Corticotropin-Releasing Factor Neuronal Activity in the Mouse Hypothalamic Paraventricular Nucleus

**DOI:** 10.3389/fnsyn.2021.804898

**Published:** 2022-01-27

**Authors:** Sarah Simmons, Ludovic D. Langlois, Mario G. Oyola, Shawn Gouty, T. John Wu, Fereshteh S. Nugent

**Affiliations:** ^1^Department of Pharmacology and Molecular Therapeutics, Uniformed Services University of the Health Sciences, Bethesda, MD, United States; ^2^Department of Gynecologic Surgery and Obstetrics, Uniformed Services University of the Health Sciences, Bethesda, MD, United States

**Keywords:** traumatic brain injury, blast injury, CRF, paraventricular nucleus, PVN, electrophysiology, neuronal activity, GABAergic synaptic transmission

## Abstract

Blast-induced mild traumatic brain injury (mbTBI) is the most common cause of TBI in US service members and veterans. Those exposed to TBI are at greater risk of developing neuropsychiatric disorders such as posttraumatic stress disorder, anxiety and depressive disorders, and substance use disorders following TBI. Previously, we have demonstrated that mbTBI increases anxiety-like behaviors in mice and dysregulates stress at the level of corticotropin-releasing factor (CRF) neurons in the paraventricular nucleus (PVN). To expand on how mTBI may dysregulate the stress axis centrally, here PVN CRF neuronal activity was evaluated using whole cell-patch clamp recordings in hypothalamic slices from sham and mbTBI adult male CRF:tdTomato mice 7 days post-injury. We found that mbTBI generally did not affect the neuronal excitability and intrinsic membrane properties of PVN CRF neurons; this injury selectively increased the frequency of spontaneous neuronal firing of PVN CRF neurons localized to the dorsal PVN (dPVN) but not ventral PVN (vPVN). Consistently, mbTBI-induced dPVN CRF hyperactivity was associated with pre- and post-synaptic depression of spontaneous GABAergic transmission onto dPVN CRF neurons suggesting that mbTBI-induced GABAergic synaptic dysfunction may underlie dPVN CRF neuronal hyperactivity and increases in dPVN CRF signaling. The present results provide the first evidence for mbTBI-induced alterations in PVN CRF neuronal activity and GABAergic synaptic function that could mediate hypothalamic CRF dysregulation following mbTBI contributing to stress psychopathology associated with blast injury.

## Introduction

Traumatic brain injury (TBI) accounts for almost 3 million hospitalizations or admissions into the emergency room in the United States with the incidence rate increasing annually (Taylor et al., 2017; GBD 2016 Traumatic Brain Injury and Spinal Cord Injury Collaborators, 2019). Blast waves are one cause of TBI, and although they are most frequently experienced by active military personnel, civilians may also suffer from blast TBIs (Hicks et al., 2010; Helmick et al., 2015; Bowen et al., 2016). Among the non-fatal injuries, most TBIs continue to be a major source of long-lasting disabilities including impairments in cognition, mood/emotional regulation, and social interactions following mTBI and contribute to the inability of affected individuals to carry out routine daily activities and to maintain important social relationships and employment (Silver et al., 2009; Wong et al., 2013; Greer et al., 2020). Additionally, up to 30% of TBI patients experience neuroendocrine dysfunction (Lieberman et al., 2001; Krahulik et al., 2010; Molaie and Maguire, 2018; Hoffman and Taylor, 2019) with a high incidence of stress axis [also known as the hypothalamic-pituitary-adrenal (HPA) axis] dysregulation with accompanying behavioral deficits (Krahulik et al., 2010; Hoffman and Taylor, 2019).

One of the major neuromodulatory stress systems that is responsive to mTBI and has a significant influence on stress-related neuronal responses and affective states, is the hypothalamic PVN CRF (also known as corticotropin releasing hormone, CRH) system (Fox et al., 2016; Kosari-Nasab et al., 2019; McCorkle et al., 2021). In addition to peripheral CRF endocrine signaling, recent studies suggest that central actions of CRF neurons may play an important role in regulating mood and stress modulation of behaviors. For example, it has been shown that biphasic responses of PVN CRF neuronal activity can mediate opposing behaviors of approaching appetitive stimuli or escaping from aversive stimuli (Kim et al., 2019). Importantly, PVN CRF neurons can regulate complex behaviors in a changing environment following stress and shift innate defensive strategies (Füzesi et al., 2016; Daviu et al., 2020).

Previously, we have shown that blast-induced mild TBI (mbTBI)-induced neuroendocrine deficits and anxiety-like behaviors across both male and female mice include dysregulation of CRF pathways in the HPA axis (neuroendocrine-projecting PVN CRF neurons) but also in extrahypothalamic regions (non-neuroendocrine-projecting PVN CRF projections; Russell et al., 2018a, b). Using the early response gene c-Fos immunoreactivity, as a marker for neuronal activation and retrograde Fluoro-Gold (FG) labeling of neuroendocrine- vs. non-neuroendocrine-projecting CRF neurons of the PVN, we demonstrated that the central and peripheral CRF pathways are susceptible to mbTBI in both male and female mice 7–10 days after the blast injury. mbTBI increased restraint stress-induced corticosterone in males while decreasing in females. mbTBI diminished the percentage of restraint-activated (c-Fos+) PVN CRF neurons in male mice while increasing in females. However, when PVN CRF projection neurons were distinguished using FG staining, we observed that mbTBI only decreased c-Fos+ immunoreactivity in PVN non-neuroendocrine CRF neurons of females in response to restraint stress without any alterations in males (Russell et al., 2018a). This suggest sex-specific modulation of PVN CRF neurons by mbTBI across different projection-specific populations of PVN CRF neurons. Additionally, CRFR2 but not CRFR1 expression was affected by mbTBI in both sexes with distinct anatomical patterns of CRFR2 gene expression in stress-related PVN limbic projections (Russell et al., 2018b). Although these initial studies demonstrate that CRF stress responses from the PVN may be altered by mbTBI, it remains unclear how mbTBI dysregulates PVN CRF neuronal function in distinct anatomical and functional sub regions of the PVN. To address this, we recorded the depolarization induced neuronal excitability and spontaneous activity of CRF neurons in ventral and dorsal sub-regions of the male mouse PVN (vPVN and dPVN). We have found that mbTBI selectively increases spontaneous CRF neuronal activity within the dPVN without any significant alteration in PVN CRF neuronal excitability across dPVN and vPVN subregions. Given that the activity of distinct CRF neurons in these anatomical subregions of the PVN is shown to be differentially modulated by some of the neurotransmitters and bioactive substances (Mukai et al., 2020) and mediates endocrine vs. non-neuroendocrine components of PVN CRF signaling (Russell et al., 2018a, b), our data suggest that mbTBI-induced persistent dPVN CRF dysfunction may primarily contribute to stress-related psychopathology following mbTBI.

## Methods

### Animals

Seven- to 9-week-old CRF:tdTomato male mice with the C57BL/6J background were generated as previously described (Russell et al., 2018a) by crossing B6(Cg)-Crh^tm(cre)Zjh^/J(CRF-IRES-Cre; RRID: IMSR_JAX:012704; stock no. 012704; The Jackson Laboratory) mice and B6.Cg-Gt(ROSA) 26Sor^tm14(CAG-tdTomato)Hze^/J (Ai14; RRID: IMSR_JAX:007914; stock no. 007914; The Jackson Laboratory) mice. The mice were same-sex housed, 2–3 per cage, and maintained at 22°C to 25°C, 50% humidity, on a 12-h light:12-h dark cycle (lights on at 0100 h) with *ad libitum* access to food and water. Mice were randomly assigned to sham or mbTBI experimental group. All animal procedures were carried out in accordance with the guidelines established by the National Institute of Health (NIH) and approved by the Uniformed Services University Institutional Animal Care and Use Committee.

### mbTBI

Mice were exposed to mbTBI under isoflurane anesthesia using the Advanced Blast Simulator (ABS; ORA Inc., Fredericksburg, VA) under isoflurane anesthesia, as previously described (Russell et al., 2018a). The ABS consisted of the driver chamber (sealed with Valmex FR 1000 PVDF lacquer 7269 membrane (Mehler Texnologies GmbH, Rheinstrasse, Germany), the transition section, and the test chamber. Increased pressure (by compressed air) in the driver chamber ruptured the acetate/mesh seal, which results in blast wave traveling through the transition section to the test chamber. The mean pressure of the blast wave was ~19 psi (19 ± 0.4 psi). Sham animals were anesthetized but were not exposed to the blast injury. Immediately after sham or injury procedures, mice were observed for righting reflex (sham = 50.6 ± 7.6 s vs. ABS = 83.4 ± 0.8 s, *p* < 0.05) and returned to the home cage for recovery. Sham and mbTBI mice were euthanized for slice preparation and electrophysiology 7 days post-injury.

### Slice Preparation

For all electrophysiology experiments, mice were anesthetized with isoflurane, decapitated, and brains were quickly dissected and placed into ice-cold artificial cerebrospinal fluid (ACSF) containing (in mM): 126 NaCl, 21.4 NaHCO_3_, 2.5 KCl, 1.2 NaH_2_PO_4_, 2.4 CaCl_2_, 1.0 MgSO_4_, 11.1 glucose, and 0.4 ascorbic acid and saturated with 95% O_2_–5% CO_2_. Briefly, coronal hypothalamic slices containing PVN were cut at 250 μm and incubated in above prepared ACSF at 34°C for at least 1 h prior to electrophysiological experiments. For patch clamp recordings, slices were then transferred to a recording chamber and perfused with ascorbic-acid free ACSF at 28°C.

### Electrophysiology

**Figure 1:**
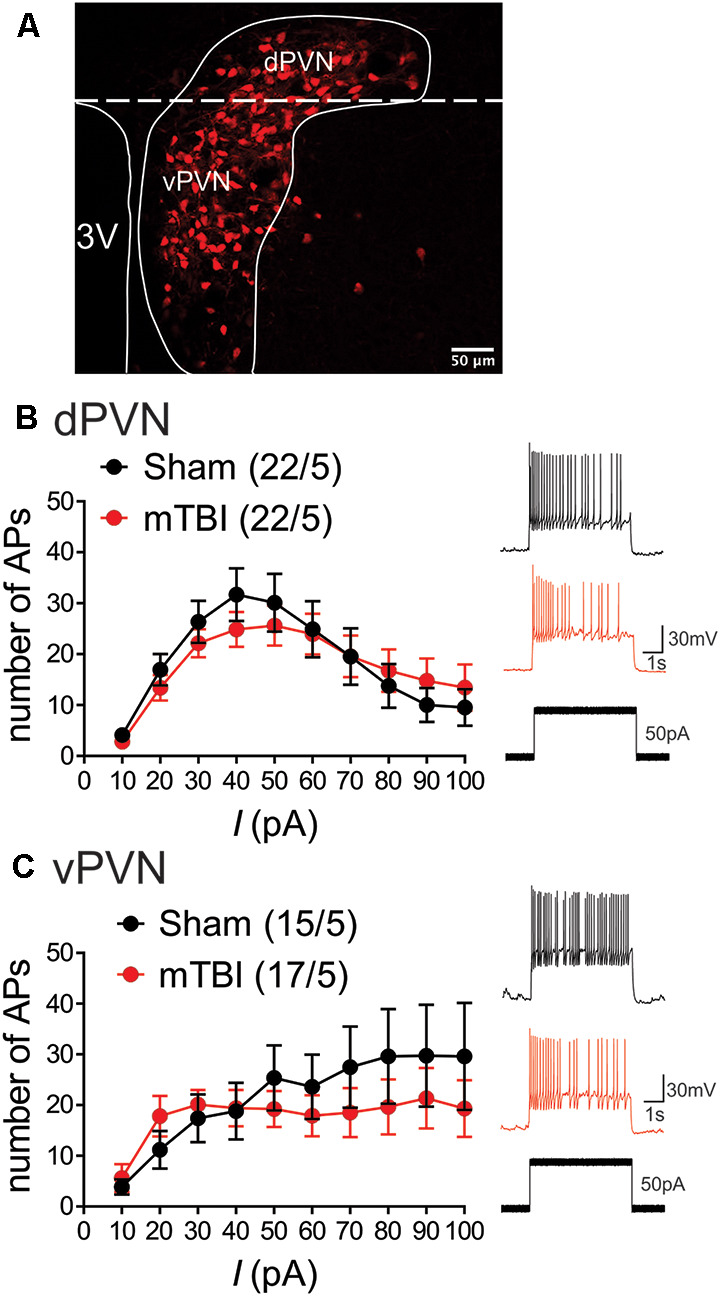
mbTBI does not alter PVN CRF neuronal excitability. **(A)** Representative image showing the expression of the red fluorescent protein TdTomato in the PVN of the CRF;TdTomato mouse. Overlay is a schematic outline of the PVN labeled as the dorsal (d) PVN and ventral (v) PVN. Average number of action potentials generated across depolarizing current steps in Sham (black) and mTBI (red) mice and representative traces in response to 50 pA stimulation across **(B)** dPVN and **(C)** vPVN CRF neurons. Group numbers presented in graph as neurons/mice.

Voltage-clamp whole-cell recordings were performed from dPVN and vPVN CRF:tdTomato neurons using patch pipettes (3–6 MOhms) and a patch amplifier (MultiClamp 700 B) under infrared-differential interference contrast microscopy. For all experiments, PVN CRF neurons were identified by the presence of tdTomato fluorescence which is a reliable indicator for CRF+ neurons within the PVN of CRF:tdTomato mice (Wamsteeker Cusulin et al., 2013). The anatomical boundary between dPVn and vPVN was defined by the mediolateral line through the dorsal edge of the third ventricle (3 V) as previously described (Mukai et al., 2020) and shown in a representative image of a hypothalamic PVN slice prepared from a CRF:tdTomato male mouse in Figure 1A. Data acquisition and analysis were carried out using DigiData 1440A, pCLAMP 10 (Molecular Devices), Clampfit, and Mini Analysis 6.0.3 (Synaptosoft, Inc.). Signals were filtered at 3 kHz and digitized at 10 kHz.

To assess PVN CRF spontaneous neuronal activity/excitability in intact synaptic transmission, cells were patch clamped with potassium-gluconate based internal solution (130 mM K-gluconate, 15 mM KCl, 4 mM adenosine triphosphate (ATP)–Na+, 0.3 mM guanosine triphosphate (GTP)–Na+, 1 mM EGTA, and 5 mM HEPES, pH adjusted to 7.28 with KOH, osmolarity adjusted to 275–280 mOsm) in slices perfused with ACSF. Electrophysiological recordings of neuronal excitability, membrane properties, and GABAergic transmission were performed as previously described (Authement et al., 2018; Simmons et al., 2020). During neuronal excitability recordings in current-clamp mode, action potential (AP) generation was assessed in response to increasingly depolarizing current steps ranging from +10 to +100pA (+10 pA ea. step) while cells were kept at −67 to −70 mV with manual direct current injection between pulses. Current steps were 5 s in duration with 25 s inter-stimulus intervals. The number of APs induced by depolarization at each intensity was counted and averaged for each experimental group at each step. Resting membrane potential (RMP) was assessed immediately after achieving whole-cell patch configuration in current clamp recordings. The hyperpolarization-activated cation current (Ih) recordings were performed in voltage-clamp in response to stepping cells from −50 mV to −100 mV (700 ms duration). Input resistance (Rin) was measured at −50 pA step (5 s duration) and calculated by dividing the change in voltage response by the current-pulse amplitude and presented as MΩ. AP threshold, fast after-hyperpolarizations (fAHP), and medium after-hyperpolarizations (mAHP) were assessed using clampfit and measured from the first AP at the current step that was sufficient to generate the first AP/s. Spontaneous neuronal activity and AP firing patterns were assessed in both cell-attached recordings in voltage-clamp mode at *V* = 0 mV and whole cell recording in current-clamp mode at *I* = 0 pA for the duration of ~1 min recording. The criteria used for designating tonic activity was set to ≥3 APs across each recording. Nearly all PVN neurons are tonically active regardless of subregion or treatment. The number of APs was counted over 1 min, and spike frequency was calculated.

Whole-cell recordings of GABA_A_R-mediated spontaneous inhibitory postsynaptic currents (sIPSC) were performed in ACSF perfused with AP-V (50 uM), DNQX (10 μM), and glycine receptor inhibitor (strychnine, 1 μM). Patch pipettes were filled with KCl internal solution (125 mM KCl, 2.8 mM NaCl, 2 mM MgCl_2_, 2 mM ATP Na+, 0.3 mM GTP-Na+, 0.6 mM EGTA, and 10 mM HEPES, pH adjusted to 7.28 with KOH, osmolarity adjusted to 275–280 mOsm). For sIPSCs, CRF neurons were voltage-clamped at −70 mV and recorded over 10 sweeps, each lasting 50 s. The cell series resistance was monitored through all the experiments and if this value changed by more than 10% or greater than 25 MΩ, data were not included.

### Statistics

Values are presented as means ± SEM. The threshold for significance was set at *p < 0.05 for all analyses. All statistical analyses of data were performed using Graphpad, Prism 9.2. For all electrophysiological data, n represents the number of recorded cells/mice. Two-way ANOVA was used across each subregion of PVN to compare neuronal excitability between sham and mbTBI across current injection. Mini Analysis software was used to detect and measure sIPSCs using preset detection parameters of IPSCs with an amplitude cutoff of 5 pA. Differences between sham and mbTBI mean and cumulative probabilities of sIPSC amplitude, charge transfer, tau decay, and frequency were analyzed using 2-tailed unpaired Student’s-*t*-tests and Kolmogorov-Smirnov tests (KS, *α* = 0.05), respectively.

## Results

### mTBI Does Not Alter PVN CRF Neuronal Excitability and Intrinsic Membrane Properties Independent of the Anatomical Locations of CRF Neurons

Given our prior observations suggesting region-specific PVN CRF dysregulation, we first investigated the effects of mbTBI on PVN CRF depolarization-induced neuronal excitability and intrinsic membrane properties in intact synaptic transmission from dPVN and vPVN CRF neurons in PVN slices from sham and mbTBI male adult CRF:tdTomato mice 7 days post-injury (Figure 1 and Table 1). We found that mbTBI did not affect neuronal excitability of dPVN or vPVN CRF neurons (Figure 1B: dPVN, *n* = 22/5 per group, 2-way ANOVA, effect of mbTBI: *F*_(1, 420)_ = 0.28, *P* = 0.59; effect of current: *F*_(9, 420)_ = 8.03, *P* < 0.0001; mbTBIxcurrent interaction: *F*_(9, 420)_ = 0.47, *P* = 0.89; Figure 1C: vPVN, *n* = 15–17/5/group, 2-way ANOVA, effect of mbTBI: *F*_(1, 300)_ = 2.11, *P* = 0.14; effect of current: *F*_(9, 300)_ = 2.32, *P* < 0.05; mbTBIxcurrent interaction: *F*_(9, 300)_ = 0.55, *P* = 0.83 ). Also we found that the effects of mbTBI on intrinsic properties were negligible where mbTBI did not significantly alter RMPs, Rin, AP threshold, mAHPs although we observed a significant reduction of fAHP amplitudes only in vPVN CRF neurons (Table 1; vPVN: fAHPs, unpaired Student’s *t*-test, *t*_(29)_ = 2.37, *P* < 0.05). Note that all of CRF neurons were tonically active and fired close to their AP thresholds. Given that the more negative RMPs in the mouse PVN are reported in CRFR1 neurons within the dPVN that are exclusively GABAergic and are rarely found to be CRF positive (Jiang et al., 2018), we believe that the CRF neurons recorded here are a different population that exhibit more positive RMPs. Our observation of unusually more depolarized RMPs for CRF neurons are also consistent with what we have observed in other spontaneously active neurons when firing in tonic mode such as lateral habenula or ventral tegmental area dopamine neurons (Shepard et al., 2020; Langlois et al., 2021). However, we acknowledge that our measurements of RMPs are always made immediately after achieving whole-cell patch configuration in current clamp recordings while the evaluation of true RMPs of spontaneously active neurons requires spike silencing. Curiously, we also found that the Ih currents recorded from PVN CRF neurons in mTBI animals, in general, had smaller amplitudes compared to those from sham animals but only Ih currents of dPVN CRF neurons recorded from mbTBI mice showed a statistically significant difference in the amplitudes in comparison to those from sham animals (Table 1; dPVN: Ih currents, unpaired Student’s *t*-test, *t*_(26)_ = 2.42, *P* < 0.05).

**Table 1:** mbTBI had negligible impact on intrinsic membrane properties of PVN CRF neurons.

	dPVN		vPVN	
Property	Sham	mTBI	Sham	mTBI
RMP	−40.9 ± 1.5, n21	−40.8 ± 1.4, n22	−42.8 ± 1.1, n16	−41.7 ± 1.3, n16
Rin	780.6 ± 97.3, n20	834.3 ± 54.45, n21	781.7 ± 85.49, n15	793.3 ± 72.22, n16
AP Threshold	−31.1 ± 0.6, n18	−32.7 ± 0.8, n21	−34.4 ± 1.1, n14	−32.6 ± 0.8, n16
fAHP	−18.2 ± 1.2, n18	−17.3 ± 1.0, n21	−17.5 ± 0.9, n15	−14.1 ± 1.2, n16
				*P < 0.05
mAHP	−43.2 ± 1.5, n18	−44.5 ± 1.5, n21	−40.0 ± 1.5, n15	−41.9 ± 1.8, n16
Ih	−20.3 ± 5.3, n13	−7.9 ± 1.2, n15	−17.2 ± 5.4, n13	−9.1 ± 1.4, n14
		*P < 0.05		

Table shows the intrinsic membrane and AP properties across dPVN and vPVN CRF neurons in sham and mTBI. Unpaired Student’s *t*-test, *p < 0.05 noted for the statistical significance in comparisons between sham and mbTBI mice.

Of note, we noticed a significant phenotypic difference between dPVN vs. vPVN CRF neuronal excitability of sham animals in response to depolarization where dPVN CRF neurons exhibited spike-frequency adaptation and higher AP thresholds compared to those from vPVN CRF neurons. In other words, while dPVN CRF neurons exhibited depolarization-induced blockade of AP generation in response to larger depolarizing step currents that could be due to inactivation of Na+ channels with somatic depolarization, vPVN CRF neurons with lower AP thresholds maintained high frequency firing and negligible spike frequency adaptation (Table 1, AP threshold, unpaired Student’s *t*-test, *t*_(30)_ = 2.69, *P* < 0.05; comparisons from sham animals in Figure 1: *n* = 15–22/5 per group, 2-way ANOVA, effect of PVN subregion: *F*_(1, 140)_ = 6.55, *P* < 0.05; effect of current: *F*_(9, 140)_ = 0.71, *P* = 0.69; PVN subregion × current interaction: *F*_(9, 140)_ = 1.26, *P* = 0.26).

### mTBI Increases dPVN but Not vPVN CRF Spontaneous Neuronal Activity

**Figure 2:**
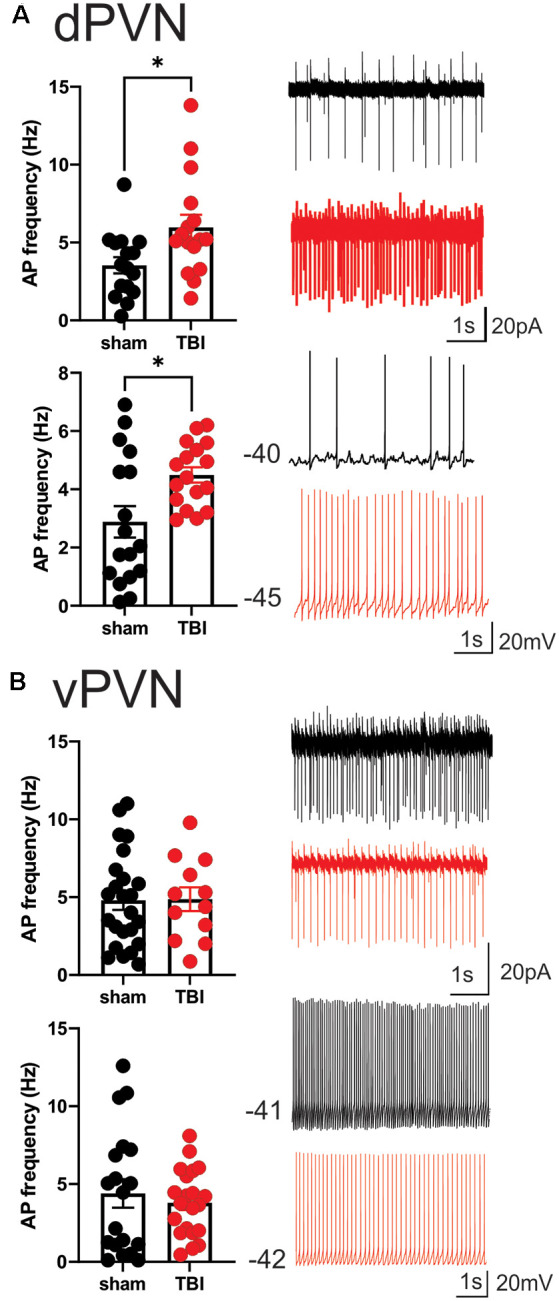
mbTBI increases spontaneous activity of dPVN CRF neurons. Representative traces, and comparison of action potential (AP) frequency under voltage-clamp (top, *V* = 0) cell-attached recordings and current clamp (bottom, *I* = 0) whole-cell recordings across sham (black) and mTBI (red) mice in **(A)** dPVN and **(B)** vPVN CRF neurons. *n* = 12–24/5/Group. Unpaired Student’s *t*-test, *p < 0.05.

The measurements of neuronal excitability are performed in response to artificial depolarization of neurons. Therefore, to assess the spontaneous activity of PVN CRF neurons without any manipulation, we recorded cell-attached voltage clamp and whole cell current clamp recordings of spontaneous neuronal activity in dPVN and vPVN neurons in PVN slices from sham and mbTBI male adult CRF:tdTomato mice 7 days post-injury. In general, we found that CRF PVN neurons are tonically active and mbTBI did not change the number of spontaneously active neurons. However, we found that only dPVN but not vPVN CRF neurons of mbTBI mice exhibited higher mean AP firing frequency compared to those from sham mice in both voltage clamp (Figure 2A, Student’s *t*-test, *t*_(30)_ = 2.53, *p* < 0.05) and current clamp recordings (Figure 2B, Student’s *t*-test, *t*_(32)_ = 2.67, *p* < 0.05) suggesting that mbTBI selectively induces dPVN hyperactivity.

### mbTBI Decrease Spontaneous GABAergic Synaptic Transmission Onto dPVN CRF Neurons

**Figure 3:**
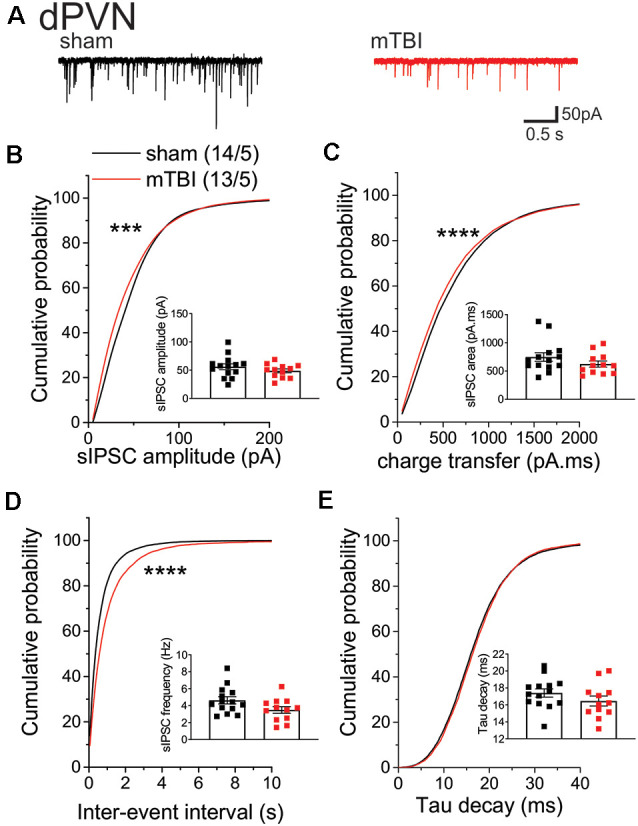
mbTBI significantly decreased spontaneous GABA_A_R-mediated synaptic transmission onto dPVN CRF neurons. **(A)** Representative traces of sham (black) and mTBI (red) spontaneous IPSPs (sIPSPs). Cumulative probability (CP) curves and group mean scatter dot-plot of sIPSPs **(B)** amplitude, **(C)** charge transfer, **(D)** inter-event interval (IEI) and **(E)** Tau decay. KS test for cumulative distribution curves, ***p < 0.001, ****p < 0.0001. Group neuron/mice numbers noted in **(B)** are identical across other sIPSP graphs.

Given that mbTBI increase dPVN neuronal activity, we then tested the effects of mbTBI on spontaneous GABAergic neurotransmission onto dPVN neurons from sham and mbTBI male adult CRF:tdTomato mice 7 days post-injury (Figure 3). Although we did not find any significant change in the group mean sIPSC amplitude (Figure 3B), charge transfer (Figure 3C), or frequency (Figure 3D) following mbTBI, we observed a significant leftward shift of the cumulative probability (CP) of sIPSC amplitude (Figure 3B, KS test, *p* < 0.001) and charge transfer (Figure 3C, KS test, *p* < 0.0001) as well as a significant rightward shift of the CP of sIPSC inter-event interval (Figure 3D, KS test, *p* < 0.0001) following mbTBI suggesting that mbTBI-induced reduction of spontaneous GABAergic neurotransmission onto dPVN CRF neurons. Note that the group mean and CP of sIPSC Tau decay did not alter by mbTBI (Figure 3D).

## Discussion

Previously, we showed that mbTBI may induce functional CRF dysregulation within neuroendocrine and non-neuroendocrine PVN CRF pathways (Russell et al., 2018a, b). As a follow up to previous studies, we were interested in a functional profile of the CRF neuronal subpopulations using electrophysiology in slice preparations from sham and mbTBI male adult CRF:TdTomato mice. We found that although mbTBI did not affect the overall neuronal excitability of dPVN and vPVN CRF neurons and its effects on intrinsic membrane properties of PVN CRF neurons were negligible, it selectively resulted in an increase in the spontaneous firing frequency of dPVN CRF neurons. dPVN CRF hyperactivity was also associated with decreased spontaneous GABAergic neurotransmission suggesting that mbTBI-induced GABAergic dysfunction could underlie the increased dPVN CRF signaling.

The parvocellular PVN CRF neurons mediate the physiological and behavioral stress responses of the HPA axis by releasing CRF into the median eminence which acts on the pituitary to release adrenocorticotropic hormone (ACTH). ACTH subsequently activates the synthesis and secretion of glucocorticoids, such as corticosterone from the adrenal glands to mediate stress responses. In addition to neuroendocrine PVN CRF neurons mostly located in the vPVN, the non-neuroendocrine, preautonomic dPVN CRF neurons project to the brainstem and spinal cord, and control sympathetic activity (Jiang et al., 2018; Mukai et al., 2020) and may be involved in mood regulation and behavioral flexibility in response to stressors, rewarding and aversive stimuli (Füzesi et al., 2016; Kim et al., 2019; Daviu et al., 2020). Therefore, our results suggest that mbTBI-induced dysregulation of non-neuroendocrine CRF pathways from the dPVN may primarily promote anxiety-like behaviors following mbTBI. We also found that mbTBI decreased spontaneous GABAergic-transmission onto dPVN CRF neurons which is most likely presynaptic in nature (less GABA release) which could support dPVN CRF hyperactivity following mbTBI. It is interesting to note that the blast injury in our paradigm did not affect glial numbers within the PVN and there was no change in Iba1+ and GFAP+ cells in the PVN of sham and mbTBI mice (data not shown) suggesting that mbTBI alters GABAergic inputs to the PVN rather than a direct insult to the PVN. Interestingly, a subpopulation of PVN parvocellular neurons in rats engage in a CRF feedback loop. These neurons express CRFR1 as well as CRF responsive HCN channels. CRF potentiates HCN channel activity and Ih currents and increases the neuronal activity of these PVN neurons (Qiu et al., 2005). A later study used CRF- and CRFR1-cre transgenic mouse lines with optogenetics circuit mapping and monosynaptic tracing provided compelling evidence for the existence of intra-PVN local CRF signaling where PVN CRF neurons form synaptic connections onto preautonomic CRFR1-expressing neurons within the PVN (Jiang et al., 2018). The almost exclusive GABAergic nature of this subset of PVN CRFR1 neurons within the dPVN is rarely found to be CRF positive and allows for a local PVN inhibitory feedback. Therefore, these GABAergic CRFR1 neurons form a microcircuit within the dPVN and may respond to the local release of CRF from PVN CRF neurons and in turn inhibit the activity of PVN CRF neurons. Interestingly, the ablation of PVN CRFR1 neurons also causes HPA axis hyperactivity (Jiang et al., 2018) suggesting that this subset of PVN neurons provide an important inhibitory synaptic braking mechanism to prevent PVN CRF hyperexcitability. Therefore, it is possible that mbTBI may remove the intrinsic inhibitory GABAergic feedback from PVN CRFR1 neurons onto dPVN CRF neurons. Specifically, mbTBI-induced decreases in presynaptic GABA release that is evident from our sIPSC recordings in dPVN CRF neurons may indicate that mbTBI results in a loss of these intrinsic GABAergic neurons or decreases their neuronal firing although we cannot exclude the possible alteration of extrinsic GABAergic synaptic inputs to PVN CRF neurons such as the median preoptic nucleus and the posterior bed nucleus of the stria terminalis by mbTBI (Bains et al., 2015). Our finding of mbTBI-induced suppression of amplitude and charge transfer of sIPSCs may also indicate that mbTBI could alter or induce postsynaptic GABAergic plasticity in dPVN CRF neurons. However, the reduction of the frequency of large sIPSCs that are AP-driven can generally impact both inter-event interval and amplitude distribution of sIPSCs, therefore mbTBI-induced changes in GABAergic transmission are most likely presynaptic in nature rather than a change in GABA_A_R conductance. PVN CRF neurons receive both glutamatergic and GABAergic synaptic inputs although the proportion of fast GABAergic synaptic transmission onto PVN CRF neurons is substantially higher than other brain regions suggesting that GABAergic inhibition mediated by GABA_A_Rs and plasticity at GABAergic synapses onto PVN CRF neurons plays an important role in PVN CRF neuronal activity and restraining baseline HPA axis CRF signaling (Bains et al., 2015). Given that mbTBI-induced glutamatergic synaptic dysfunction in PVN CRF neurons is also likely to occur, it would be worthwhile to investigate input-specific glutamatergic and GABAergic plasticity and the shift in the excitation/inhibition balance across specific PVN populations. Interestingly, we also found that mbTBI decreases Ih currents in dPVN CRF neurons which may be a homeostatic response to dPVN CRF hyperactivity following mbTBI or an indication of intrinsic plasticity. We acknowledge that our present results in male mice set a good foundation for future mbTBI studies in females where we expect to find sex- and subregion-specific differences in PVN CRF neuronal activity. Overall, our data suggest that mbTBI has differential effects on distinct neuroanatomical CRF pathways where it induces persistent GABAergic synaptic dysfunction in dPVN CRF neurons to promote dPVN CRF hyperactivity thereby selectively increasing hypothalamic as well as extrahypothalamic CRF signaling and promoting anxiety-like behaviors. Given the emerging role of extrahypothalamic PVN CRF signaling, our future studies will also consider central PVN CRF projections to stress-related brain circuits that may contribute to stress psychopathology associated with this model of mTBI.

## Data Availability Statement

The original contributions presented in the study are included in the article, further inquiries can be directed to the corresponding author/s.

## Ethics Statement

The animal study was reviewed and approved by Uniformed Services University Institutional Animal Care and Use Committee.

## Author Contributions

FN and TJW were responsible for the study concept and design. SS, LL, MO, and SG contributed to the acquisition of animal data. LL, FN, and SS assisted with data analysis and interpretation of findings. FN, SS, LL, and TJW wrote the manuscript. All authors critically reviewed content and approved final version of manuscript for submission. All authors contributed to the article and approved the submitted version.

## Conflict of Interest

The authors declare that the research was conducted in the absence of any commercial or financial relationships that could be construed as a potential conflict of interest.

## Publisher’s Note

All claims expressed in this article are solely those of the authors and do not necessarily represent those of their affiliated organizations, or those of the publisher, the editors and the reviewers. Any product that may be evaluated in this article, or claim that may be made by its manufacturer, is not guaranteed or endorsed by the publisher.
